# Early life adversity impaired dorsal striatal synaptic transmission and behavioral adaptability to appropriate action selection in a sex-dependent manner

**DOI:** 10.3389/fnsyn.2023.1128640

**Published:** 2023-04-05

**Authors:** Gregory de Carvalho, Sheraz Khoja, Mulatwa T. Haile, Lulu Y. Chen

**Affiliations:** ^1^Department of Anatomy & Neurobiology, School of Medicine, University of California, Irvine, Irvine, CA, United States; ^2^UCI-Conte Center, UCI-NIMH, University of California, Irvine, Irvine, CA, United States

**Keywords:** early life adversity, corticostriatal circuits, synaptic transmission, action-outcome contingency, outcome devaluation, reversal learning, behavioral flexibility

## Abstract

Early life adversity (ELA) is a major health burden in the United States, with 62% of adults reporting at least one adverse childhood experience. These experiences during critical stages of brain development can perturb the development of neural circuits that mediate sensory cue processing and behavioral regulation. Recent studies have reported that ELA impaired the maturation of dendritic spines on neurons in the dorsolateral striatum (DLS) but not in the dorsomedial striatum (DMS). The DMS and DLS are part of two distinct corticostriatal circuits that have been extensively implicated in behavioral flexibility by regulating and integrating action selection with the reward value of those actions. To date, no studies have investigated the multifaceted effects of ELA on aspects of behavioral flexibility that require alternating between different action selection strategies or higher-order cognitive processes, and the underlying synaptic transmission in corticostriatal circuitries. To address this, we employed whole-cell patch-clamp electrophysiology to assess the effects of ELA on synaptic transmission in the DMS and DLS. We also investigated the effects of ELA on the ability to update action control in response to outcome devaluation in an instrumental learning paradigm and reversal of action-outcome contingency in a water T-maze paradigm. At the circuit level, ELA decreased corticostriatal glutamate transmission in male but not in female mice. Interestingly, in DMS, glutamate transmission is decreased in male ELA mice, but increased in female ELA mice. ELA impaired the ability to update action control in response to reward devaluation in a context that promotes goal-directedness in male mice and induced deficits in reversal learning. Overall, our findings demonstrate the sex- and region-dependent effects of ELA on behavioral flexibility and underlying corticostriatal glutamate transmission. By establishing a link between ELA and circuit mechanisms underlying behavioral flexibility, our findings will begin to identify novel molecular mechanisms that can represent strategies for treating behavioral inflexibility in individuals who experienced early life traumatic incidents.

## 1. Introduction

Early life adversity (ELA) is commonly reported in the United States, with 62% of adults reporting at least one adverse childhood experience (Merrick et al., 2019). Adversity during a critical developmental period can negatively impact neural circuits and has long been associated with neuropsychiatric disorders later in life, with many studies reporting behavioral, cognitive, and emotional impairments (Kessler et al., 1994; Dias-Ferreira et al., 2009; Wang et al., 2011; Baram et al., 2012; Gershon et al., 2013; Molet et al., 2016; Davis et al., 2017; Bolton et al., 2018a; Glynn and Baram, 2019; Gasser et al., 2020; He et al., 2021; Levis et al., 2022; Xu et al., 2022). Investigating the molecular mechanisms underlying ELA-induced cognitive and emotional deficits is critical to laying the foundation for novel strategies in therapeutic intervention.

ELA-induced neurodevelopmental and behavioral deficits were initially documented by using the maternal separation (MS) model in rodents, in which the mother was separated from her pups intermittently over a specific period of time (Russell, 1973; Hall, 1998; Ladd et al., 2000). However, these models lacked translational validity since mothers who are diagnosed with psychiatric disorders (depression, schizophrenia, or drug addiction) can be negligent in their children’s care or abusive towards them, even though they are still present in the household.

The limited bedding and nesting (LBN) paradigm induces an impoverished environment that negatively impacts the quality of maternal care, leading to fragmented and unpredictable nurturing behaviors (Gilles et al., 1996; Brunson et al., 2005; Ivy et al., 2008). Multiple investigations have reported the effects of ELA on cognitive (spatial, working, and contextual memory) and emotional behaviors using the LBN paradigm (Ivy et al., 2008, 2010; Naninck et al., 2015; Arp et al., 2016; Bath et al., 2017; Davis et al., 2017; Xu et al., 2022). These behavioral deficits were linked to alterations in developmental neurogenesis and structural abnormalities, especially in the hippocampus, hypothalamus, and paraventricular nucleus (Ivy et al., 2010; Korosi et al., 2010; Naninck et al., 2015; Lapp et al., 2020; Levis et al., 2022).

While novel and exciting insights have been achieved in determining the effects of ELA in the hippocampus, very little attention has been given to other anatomically and functionally interacting brain regions such as the dorsal striatum. The dorsal striatum is densely innervated by cortical regions such as the sensorimotor, motor, and cingulate cortex (Hunnicutt et al., 2016; Hadjas et al., 2020) and regulates the balance between goal-directed and habitual actions (Yin and Knowlton, 2004; Yin et al., 2004, 2005a,b; 2006; Gremel and Costa, 2013; Gremel et al., 2016). To date, only two studies using the LBN paradigm have reported neurodevelopmental deficits in the dorsal striatum and perturbations in associated behavioral functions, including decision-making (He et al., 2021; Xu et al., 2022). ELA delayed dendritic differentiation in the dorsolateral (DLS), but not in the dorsomedial (DMS) striatum, in mice at P16 (He et al., 2021); which was later accompanied by a significant increase in thin and mushroom-type spines in DLS at P120 (Xu et al., 2022). At the behavioral level, ELA-induced mice shifted from goal-directed to habitual control upon extensive instrumental training in a context that promotes goal-directedness (Xu et al., 2022). However, this study did not address the adaptability of ELA-induced mice to alternate between goal-directed and habitual actions under differing contingencies of reward delivery that can bias differential action control. Additionally, previous studies have not investigated the ability of ELA mice to disengage from preferred behavioral patterns and learn new ones in behavioral paradigms that assess intradimensional shifts in behavior, such as reversal learning tasks. Moreover, the functional impact of ELA on synaptic activity in brain regions linked to the balance between goal-directed and habitual actions, such as the dorsal striatum, has not been thoroughly investigated.

To this end, our study begins to explore three questions that remain unanswered: (i) whether ELA impacts corticostriatal glutamate transmission in a manner that explains behavioral impairments; (ii) whether ELA affects the flexibility of mice to adopt different action selection strategies; and (iii) whether ELA impairs reversal learning. We also aim to address these questions in a sex-dependent manner on the basis of a plethora of studies showing differences in cognitive and emotional behaviors between male and female mice subjected to ELA (Colich et al., 2017; Ellis and Honeycutt, 2021; Bondar et al., 2018; Ruigrok et al., 2021a,b). The sex-dependent differences in behaviors can be attributed to discrepancies in maternal care, where male mice receive more care than their female siblings (Oomen et al., 2009; van Hasselt et al., 2012). Additionally, ELA has also been reported to induce behavioral and structural brain abnormalities in a sex-dependent manner in the clinical population (Frodl et al., 2010; Alastalo et al., 2013; Zoladz et al., 2022).

We performed whole-cell patch-clamp recordings in medium spiny neurons (MSNs) of DLS and DMS. We assessed the ability of LBN mice to alternate between different action-selection strategies under different contingencies of reinforcement delivery, in a context-dependent operant conditioning paradigm. Finally, we employed a water T-maze paradigm to test the impact of ELA on reversal learning capacity. Electrophysiology recordings revealed that ELA broadly decreased excitatory transmission of cortical inputs to the dorsal striatum of males, whereas only the DMS in females was affected. Failed execution of goal-directed action strategies was observed in male LBN mice, without any impairments in action selection strategies in female LBN mice. LBN mice exhibited reversal learning deficits. Together, our results suggest that LBN can disrupt the ability to integrate the value of rewards with action-selection strategies in a sex-dependent manner and to learn new actions upon contingency reversal in a sex-independent manner. Region-specific and sex-dependent impairments observed in excitatory synaptic transmission constitute a plausible synaptic mechanism underlying behavioral inflexibility in LBN mice. These findings can lay the groundwork for therapeutic strategies for behavioral inflexibility in individuals who experienced adverse childhood experiences.

## 2. Methods

### 2.1. Animals

Male and female C57BL/6J mice for breeding and generation of litters for control (CTL) and LBN group were purchased from Jackson laboratories (Bar Harbor, ME). Breeding pairs were maintained on a 12/12 h light/dark cycle with *ad libitum* access to water and a high-fat chow. All procedures were conducted in accordance with the National Institute of Health Guide for the Care and Use of Laboratory Animals and protocols approved by the Institutional Animal Care and Use Committee of the University of California Irvine, including efforts to minimize suffering and the number of animals used.

### 2.2. LBN paradigm

LBN was carried out as previously described (Rice et al., 2008). On postnatal day 2 (P2), the dams and pups were transferred to cages fitted with an iron mesh (in cm: 15.24 wide × 33.02 long, 2.54 tall) which was placed at approximately 2.5 cm above the cage floor to allow for the collection of droppings. The cage floor was sparsely covered with bedding materials and half of a nestlet was provided. For the CTL group, the dams and pups were transferred to cages with a normal amount of bedding material and one full nestlet. The cages were left undisturbed from P2 till P9. At P10, the pups and the dams from CTL and LBN group were transferred to cages with a normal amount of bedding material and one nestlet. Mice were weaned at P21 and were used for behavioral tests at 2–4 months of age and electrophysiology experiments at 2–5 months of age. Separate cohorts of mice were used for electrophysiological and behavioral experiments. For the behavioral experiments, separate cohorts of mice were used for instrumental learning paradigm and water T-maze.

### 2.3. Instrumental learning paradigm

#### 2.3.1. Behavioral apparatus

The balance between goal-directed and habitual behaviors was investigated by using two identical operant chambers (Med-Associates, St. Albans, VT), enclosed in sound attenuating boxes that were differentiated by contextual cues (15 mm wide multi-colored washi tape that was vertically and horizontally aligned on chamber walls to give a checkerboard-like pattern or clear plexiglass chamber walls). Each chamber was equipped with a pellet dispenser that delivered 20 mg food pellets (Bio-Serv, Flemington, NJ) into a recessed food magazine, two retractable levers on either side of the food magazine, a house light, a stainless-steel grid floor and an 8 input/16 output connection panel that serves as an interface between the chambers, and a computer that runs the MED-PC V software (Med-Associates, St. Albans, VT) to operate the experimental paradigms and record lever pressing behavior. Prior to training, mice were handled, and food restricted to 85%–90% of their baseline body weight. Their body weights were maintained within this range during the entirety of the instrumental learning paradigm.

### 2.3.2. Context-dependent training paradigm

Behavioral training and testing were conducted as previously described (Gremel et al., 2016). Briefly, following 3 days of handling and food restriction, mice underwent a training session on days 4–11. Each training session commenced with switching on of the house light and extension of a single lever and ended following completion of the instrumental learning task or after 60 min with the lever withdrawn and switching off of the house light. The lever position, order of context exposure, and schedule order were constant for each mouse throughout the entire training period and outcome devaluation test and counterbalanced between mice. On the first day, mice were trained to associate the food magazine with reinforcement presentation in two separate contexts, using a random time (RT) schedule in which a food pellet was delivered on an average of 60 s in the absence of levers. This session would continue for a total period of 15 min or when 15 reinforcers had been delivered. Following the RT schedule, on the same day, mice were trained to press a single lever under a continuous ratio of reinforcement (CRF1) in each context, wherein every lever press was reinforced with one pellet. Mice underwent CRF1 on days 4–5, with the number of earned reinforcers increasing on each day (i.e., 5 reinforcers on day 4, 15 and then 30 reinforcers on day 5). All the CRF sessions continued for a total period of 60 min or when the maximum number of earned reinforcers was dispensed. Following the acquisition of lever pressing behavior, mice underwent random interval (RI) and random ratio (RR) schedules of reinforcement that were differentiated by contexts from days 6–11. On days 6 and 7, mice were trained to press the lever under RI30, wherein the passing of an interval period of 30 s had a 15% probability of delivering a food pellet and RR10, in which every lever press had a 10% chance of delivering a food pellet. This was followed by RI60 and RR20 on days 8–11. All the RI and RR sessions ended once 60 min had elapsed or 15 reinforcers were dispensed. Following the RI and RR sessions, mice were provided with 1 h access to a 20% sucrose solution in their home cage as a satiety control for the outcome devaluation test.

### 2.3.3. Outcome devaluation test

This procedure was undertaken on days 12 and 13 of the post training phase. On the valued day, mice had 1 h *ad libitum* access to a 20% sucrose solution in their home cage, followed by brief, non-reinforced test sessions for 5 min in both RI and RR contexts. On the devalued day, mice had 1 h *ad libitum* access to 20 mg food pellets that they previously acquired by lever pressing, followed by brief, non-reinforced test sessions for 5 min in both the RI and RR contexts. The order of devaluation was counterbalanced between mice.

### 2.3.4. Water T-maze

Spatial learning and reversal learning was assessed by the water T-maze paradigm (Guariglia and Chadman, 2013). The T-maze was filled with 20°C (±1°C) water to a depth of 13 cm which is 1 cm above the surface of the platform. Water was made opaque by using non-fat dry milk. On the first day (pre-training phase), mice were allowed to swim freely in the T-maze without the platform for 60 s and the first arm that the mice entered was recorded. On all the training days, the platform was placed in the arm opposite to the one chosen during pre-training. The training session began 24 h after the pre-training phase in which the mice were given 10 trials per day with 7–10 min of rest in between each trial. Mice were placed in the start arm and given 60 s to find the platform. Once the platform was found, the mice were forced to stay on the platform for 5 s. If they were not able to find the platform within 60 s, they were gently guided to the platform and forced to stay on it for 10 s. Mice were charged with errors if: (1) they left the start arm and entered the arm that does not contain the platform or (2) they entered the arm with the platform but left that arm without staying on the platform. A trial is considered successful when the mouse leaves the start arm and enters the arm with the platform and stays on it. The training session ended and the reversal learning phase began when the mice reached the criteria of eight successful trials or greater for two consecutive days. For the reversal learning phase, the platform was placed in the arm opposite to the one chosen during the training session, and the same procedure for charging errors and determining a successful trial was followed as described above. The number of incorrect arm entries and the success rate (percent of trials without an error) were calculated for the training and reversal learning phase.

### 2.4. Electrophysiology

#### 2.4.1. Slice preparation

Mice were deeply anesthetized with isoflurane and quickly decapitated. Acute coronal slices (300 μm) were obtained using a vibratome (Leica V1200S) in an ice-cold N-Methyl D-Glucamine (NMDG) cutting solution containing (in mM): 110 NMDG, 20 HEPES, 25 glucose, 30 NaHCO_3_, 1.2 NaH_2_PO_4_, 2.5 KCl, 5 Na-ascorbate, 3 Na- pyruvate, 2 Thiourea, 10 MgSO_4_-7 H_2_O, and 0.5 CaCl_2_ (305–310 mOsm, pH 7.4). Slices equilibrated in a homemade chamber for 25–30 min (31°C) and an additional 45 min in room temperature aCSF containing (in mM): 119 NaCl, 26 NaHCO_3_, 1 NaH_2_PO_4_, 2.5 KCl, 11 Glucose, 10 Sucrose, 1.3 MgSO_4_-7 H_2_O, and 2.5 CaCl_2_ (290–300 mOsm, pH 7.4), before being transferred to a recording chamber. All solutions were continuously bubbled with 95% O_2_/5%CO_2_.

#### 2.4.2. Whole-cell patch-clamp

Whole-cell patch-clamp recordings were obtained from MSNs in the DLS or DMS. Data were collected with a Multiclamp 700B, Digidata 1550B, and Clampex 11 (pClamp; Molecular Devices, San Jose, CA). All recordings were acquired in voltage clamp at 31°C and were low pass filtered at 2 kHz and digitized at 10 kHz. Membrane voltage was held at −70 mV for all measures unless otherwise specified. Recording pipette was filled with internal solution containing (in mM): 135 CsMeSO_4_, 8 CsCl, 10 HEPES, 0.25 EGTA, 5 Phosphocreatine, 4 MgATP, 0.3 NaGTP, and 1 mg/ml NeuroBiotin (295–305 mOsm, pH 7.4 with CsOH). Picrotoxin (50 μM) was added to aCSF, and only excitatory postsynaptic currents (EPSCs) were recorded. All pipettes (3–4 MΩ) were pulled from borosilicate glass (Narishige PC-100). Access resistance (Ra) was monitored throughout the recording and cells that increased Ra by > 20% were discarded. Cells were visualized under infrared direct interference contrast (Olympus BX51WI; Olympus, Philadelphia). The current study did not distinguish between D1 and D2 MSNs. Recordings in either DMS (DMS-MSN) or DLS (DLS-MSN) were stimulated using a tungsten bipolar stereotrode (MicroProbes, Maryland), placed dorsal to the recorded cell and at the border of the corpus callosum and striatum. Distance of the stimulating electrode from the recorded cell did not exceed 200 μm and was no less than 150 μm. Electrical pulse durations were limited to a 50–100 μs range. For all recordings, electrical stimulation was delivered every 10 s (0.1 Hz). **Input/Output (I/O)** relationship between stimulation intensity and EPSC amplitude was measured by stimulating the cortical inputs in increments of 10 μA, starting at 0 μA and terminating at 200 μA. Slices were not stimulated past 200 μA to ensure slice health and allow for recordings of multiple cells on the same slice. **Paired-Pulse Ratio (PPR)** was measured by electrically evoking EPSCs with two pulses at an inter-pulse interval of 25 ms (40 Hz). Ratio was determined by dividing the amplitude of the second EPSC by the amplitude of the first EPSC. **α-amino-3-hydroxy-5-methyl-4-isoxazole propionic acid/ N-Methyl-D-aspartate** (**AMPA/NMDA) ratio** was measured by evoking EPSCs while holding the cell at −70 mV then at +40 mV. Peak amplitudes at −70 mV were used as the measure for the AMPA component. 150 ms after the onset of the response at +40 mV was used as the measure for the NMDA component. Ratio was calculated by dividing the AMPA component by the NMDA component. Total recording time for each cell was 15 min minimum to allow for adequate dialyzing of NeuroBiotin in the cytosolic space. After recording, slices were post-fixed in 4% paraformaldehyde overnight at 4°C. Post-fixed slices were then transferred to a 0.1 M phosphate buffer until histology processing.

#### 2.4.3. Immunohistochemistry

Visualization of NeuroBiotin-filled cells was performed as previously described (Gunn et al., 2019). Briefly, slices used for whole-cell patch-clamp (300 μm) were sectioned to 40 μm thickness using a Microm HM440E sliding microtome (Fisher Scientific, Pittsburgh), and stained with Streptavidin Alexa Fluor 568 conjugate (1:200: Molecular Probes, Oregon) for visualization of NeuroBiotin-filled cell, using an epifluorescent microscope (BZ-X800, Keyence Corporation, Illinois). Images were taken using a 60× oil immersion objective, and a z-stack (0.5 μm step) spanning the entire cell was applied; range of images taken per cell was 25–30 images. Imaged cells were visually inspected to confirm cell identity based on morphological characteristics. Round soma (around 10–20 μm in diameter) and well-defined dendritic spines were used as the criteria for cell inclusion as these have been shown to be characteristics of MSNs (Tepper et al., 1998). Cells that did not fit these criteria were excluded from analysis.

### 2.5. Statistical analyses

The behavioral experiments were analyzed by repeated measures (RM) ANOVA using day (for instrumental performance and water T-maze) or devaluation state (for outcome devaluation test) as the repeated variable and LBN condition as the independent variable, followed by Sidak’s *post-hoc* test for multiple comparisons. To investigate the within-subject distribution of lever presses between valued and devalued states, the number of lever presses for valued and devalued states was normalized to the total number of lever presses for both states (valued + devalued) in each context. This was followed by conducting a one sample t-test to determine whether the normalized value of lever presses in each state significantly differed from a hypothetical mean of 0.5, which normally reflects equal distribution of lever presses between valued and devalued states. A devaluation index was calculated by using the formula: [(valued presses − devalued presses)/total number of lever presses] in each context. I/O curve measures were analyzed by repeated measures ANOVA with stimulation intensity as the repeated variable and LBN condition as the independent variable. AMPA/NMDA and PPR measures were analyzed using a Student’s *t*-test to compare averages between groups. Sexes were separated for instrumental learning, outcome devaluation, and electrophysiology experiments but were combined for water T-maze. Two-way ANOVA analysis was conducted to assess effects of sex on PPR, AMPA/NMDA ratio, spatial navigation and reversal learning using day as repeated variable and sex or LBN condition as two independent variables. All data were expressed as mean ± standard error of the mean (SEM). Number of neurons/mouse or number of mice were indicated inside bars or by their respective plots. Significance was set at *P* < 0.05. All data were analyzed by GraphPad Prism software (San Diego, CA).

## 3. Results

### 3.1. Fragmented maternal care early in life decreased cortical glutamate release in DLS and DMS of male mice

**Figure 1:**
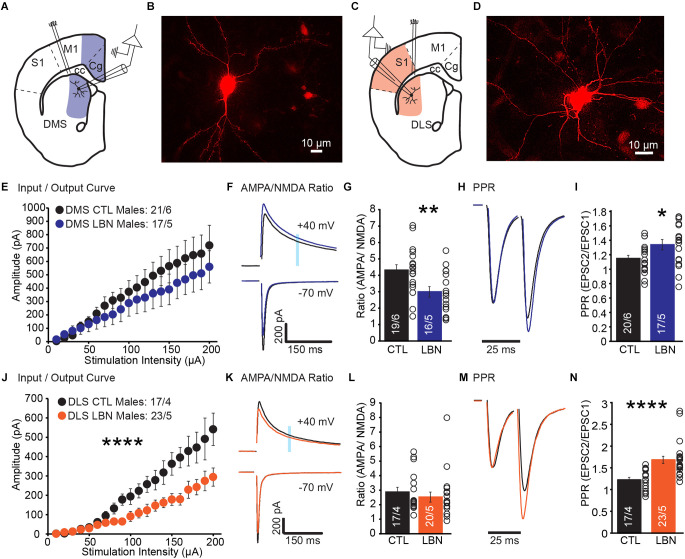
Male LBN mice exhibited decreased cortical glutamate release probability in the DLS and DMS. **(A)** Recording schematic for whole-cell patch-clamp experiments in the DMS. **(B)** Representative image of a recorded MSN in DMS; NeuroBiotin-filled cells were processed with an Alexa Fluor 568 conjugated streptavidin for morphological identification of MSN. **(C)** Recording schematic for whole-cell patch-clamp experiments in the DLS. **(D)** Representative image of a recorded MSN in DLS; NeuroBiotin-filled cells were processed with an Alexa Fluor 568 conjugated streptavidin for morphological identification of MSN. **(E)** Summary graph of input/output curve showing no changes of amplitudes in response to increasing stimulation intensities in the DMS-MSN of male LBN mice. **(F,G)** LBN altered relative contribution of AMPA and NMDA receptors to overall EPSCs in the DMS-MSN. **(F)** Representative traces of AMPA currents (−70 mV) and NMDA currents (+40 mV); shaded area denotes the region used to measure the NMDA component of EPSCs. **(G)** Summary graph of AMPA and NMDA ratio showing a decrease in ratio for male LBN mice. **(H,I)** Male LBN mice exhibited decreased probability of glutamate release in the DMS-MSN. **(H)** Representative traces of paired-pulse recordings; inter-stimulus interval was fixed at 25 ms (40 Hz) and traces were normalized to the first EPSC. **(I)** Summary graph of PPR showing a increase in ratio for male LBN mice. **(J)** Summary graph of input/output curve showing a significant decrease of amplitudes in response to increasing stimulation intensities in the DLS-MSN of male LBN mice. **(K,L)** LBN changed relative contribution of AMPA and NMDA receptors to overall EPSCs in the DLS-MSN. **(K)** Representative traces of AMPA currents (−70 mV) and NMDA currents (+40 mV); shaded area denotes the region used to measure the NMDA component of EPSCs. **(L)** Summary graph of AMPA and NMDA ratio showing no changes in ratio for male LBN mice. **(M,N)** Male LBN mice exhibited decreased probability of glutamate release in DLS-MSN. **(M)** Representative traces of paired-pulse recordings; inter-stimulus interval was fixed at 25 ms (40 Hz) and traces were normalized to the first EPSC. **(N)** Summary graph of PPR showing an increase in ratio for male LBN mice. Data is represented as means ± SEM. Number of neurons/mice are listed inside the bar graphs. Each open circle in the summary graphs represents the average of each recorded cell. Statistical assessments were performed by unpaired two-tailed Student’s t-test **(G,I,L,N)** and RM two-way ANOVA **(E,J)** by comparing male LBN to CTL mice with *p < 0.05, **p < 0.01, ****p < 0.0001.

A previous study showed that densities of dendritic spine and postsynaptic density protein-95 (PSD-95) were increased in DLS-MSN, but not DMS-MSN, of LBN male mice (Xu et al., 2022), suggesting that LBN mice had more synapses in DLS-MSN than CTL mice. However, direct measurements of synaptic transmission in MSNs post-ELA have not been investigated. To address this, we performed whole-cell patch-clamp recordings from DLS-MSN and DMS-MSN (Figures 1A–D). We recorded EPSCs and measured overall glutamate transmission from cortical synapses (I/O Curve), contribution of AMPA and NMDA receptors to overall EPSCs (AMPA/NMDA Ratio), and presynaptic probability of neurotransmitter release (PPR).

In DMS, male LBN mice showed no changes in I/O curve (RM 2-way ANOVA: *F*_(19,665)_ = 1.01, *p* = 0.45), indicating that overall cortical excitatory transmission to DMS-MSNs remained unchanged following ELA (Figure 1E). The ratio between AMPA and NMDA currents was significantly decreased in LBN mice (Figures 1F,G), suggesting that ELA caused changes in the relative contribution of AMPA and NMDA receptors to overall EPSCs (CTL = 4.32 ± 0.32, LBN = 2.99 ± 0.32; *p* < 0.01; two-tailed Student’s *t*-test). Closer examination of AMPA and NMDA currents suggest that the difference in ratio could be due to smaller amplitudes of AMPA currents (CTL: -918.1 pA; LBN: -766.1 pA) and larger NMDA currents (CTL: 212.3 pA; LBN: 251.1 pA), albeit comparisons of raw amplitudes for each current between CTL and LBN mice were not significantly different. Finally, PPR was significantly increased (Figures 1H,I), indicating a decrease in the probability of glutamate release (CTL = 1.14 ± 0.04, LBN = 1.34 ± 0.07; *p* < 0.05; two-tailed Student’s *t*-test).

In DLS, male LBN mice showed a significant impairment in I/O curve (RM 2-way ANOVA: *F*_(19,722)_ = 9.465, *p* < 0.001), indicating that overall cortical excitatory transmission to DLS-MSNs was impaired following LBN (Figure 1J). However, a decrease in I/O curve does not inform us if the impairment is pre- or post-synaptic. AMPA/NMDA ratio was measured to investigate if ELA induced any postsynaptic alterations in DLS-MSNs. The AMPA/NMDA ratio did not differ between LBN and CTL mice in DLS (Figures 1K,L), suggesting that the relative contribution of AMPA and NMDA receptors to EPSCs was not affected by LBN (CTL = 2.87 ± 0.33, LBN = 2.52 ± 0.34; *p* = 0.47; two-tailed Student’s *t*-test). Next, we asked if the results observed in DLS were due to impairments in the presynaptic probability of glutamate release. PPR recordings revealed a significant increase (Figures 1M,N) in ratio for LBN mice in the DLS (CTL = 1.23 ± 0.06, LBN = 1.69 ± 0.08; *p* < 0.0001; two-tailed Student’s *t*-test).

Together, these results show a region-specific impairment caused by ELA in sub-regions within the dorsal striatum. While the probability of glutamate release decreased in both regions, only DMS showed changes in AMPA/NMDA ratio. In DLS, it is likely that the synaptic impairment is localized predominately in presynaptic compartments, while in DMS it is likely that pre- and post-synaptic alterations occurred post-ELA.

### 3.2. Fragmented maternal care early in life increased cortical glutamate release in DMS of female mice

**Figure 2:**
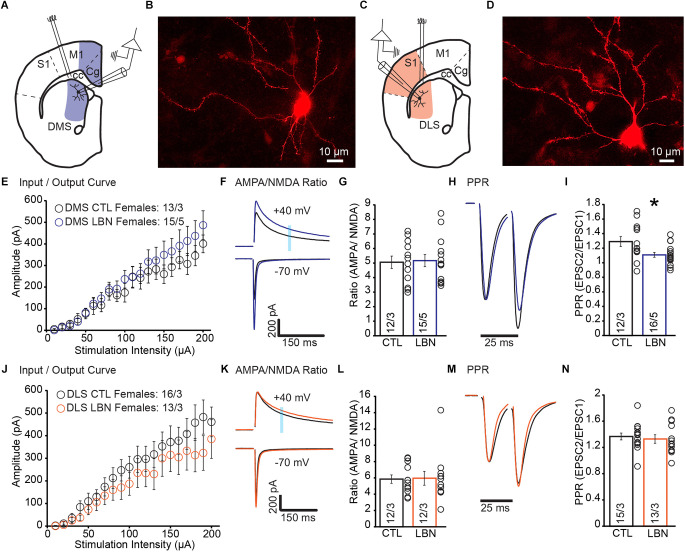
Female LBN mice exhibited increased cortical glutamate release probability in the DMS. **(A)** Recording schematic for whole-cell patch-clamp experiments in the DMS. **(B)** Representative image of a recorded MSN in DMS; NeuroBiotin-filled cells were processed with an Alexa Fluor 568 conjugated streptavidin for morphological identification of MSN. **(C)** Recording schematic for whole-cell patch-clamp experiments in the DLS. **(D)** Representative image of a recorded MSN in DLS; NeuroBiotin-filled cells were processed with an Alexa Fluor 568 conjugated streptavidin for morphological identification of MSN. **(E)** Summary graph of input/output curve showing no changes of amplitudes in response to increasing stimulation intensities in the DMS-MSN of female LBN mice. **(F,G)** LBN has no effect on the relative contribution of AMPA and NMDA receptors to overall EPSC in the DMS-MSN. **(F)** Representative traces of AMPA currents (−70 mV) and NMDA currents (+40 mV); shaded area denotes the region used to measure the NMDA component of EPSCs. **(G)** Summary graph of AMPA and NMDA ratio showing no changes in ratio for female LBN mice. **(H,I)** Female LBN mice exhibited increased probability of glutamate release in the DMS-MSN. **(H)** Representative traces of paired-pulse recordings; inter-stimulus interval was fixed at 25 ms (40 Hz) and traces were normalized to first EPSC. **(I)** Summary graph of PPR showing a decrease in ratio for female LBN mice. **(J)** Summary graph of input/output curve showing no changes of amplitudes in response to increasing stimulation intensities in the DLS-MSN of female LBN mice. **(K,L)** LBN had no effect on the relative contribution of AMPA and NMDA receptors to overall EPSC in the DLS-MSN. **(K)** Representative traces of AMPA currents (−70 mV) and NMDA currents (+40 mV); shaded area denotes the region used to measure the NMDA component of EPSCs. **(L)** Summary graph of AMPA and NMDA ratio showing no changes in ratio for female LBN mice. **(M,N)** Female LBN mice had no changes in the probability of glutamate release in the DLS-MSN. **(M)** Representative traces of paired-pulse recordings; inter-stimulus interval was fixed at 25 ms (40 Hz) and traces were normalized to first EPSC. **(N)** Summary graph of PPR showing no changes in ratio for female LBN mice. Data is represented as means ± SEM. Number of neurons/mice are listed inside the bar graphs. Each open circle in the summary graphs represents the average of each recorded cell. Statistical assessments were performed by RM two-way ANOVA **(E,J)** and unpaired two-tailed Student’s *t*-test **(G,I,L,N)** by comparing female LBN to CTL mice with *p < 0.05.

There is a growing body of work showing that ELA induced differential effects in males and females (Naninck et al., 2015; Arp et al., 2016; Bath et al., 2017; Colich et al., 2017; Bondar et al., 2018; Ellis and Honeycutt, 2021; Ruigrok et al., 2021a,b). Thus, we hypothesized that the impact of ELA on corticostriatal synapses would show sex-specific impairments. We recorded EPSCs and measured I/O curve, AMPA/NMDA ratio, and PPR (Figures 2A–D).

In DMS of female mice, I/O curve (RM 2-way ANOVA: *F*_(19,494)_ = 0.97, *p* = 0.50) and AMPA/NMDA ratio (CTL = 5.05 ± 0.43, LBN = 5.16 ± 0.42; *p* = 0.85; two-tailed Student’s *t*-test) showed no significant changes between CTL and LBN mice (Figures 2E–G). PPR was significantly decreased in female LBN mice (CTL = 1.29 ± 0.07, LBN = 1.11 ± 0.03; *p* < 0.05; two-tailed Student’s *t*-test), indicating an increase in the probability of glutamate release after LBN (Figures 2H,I).

In DLS, female LBN mice showed no significant impairments in I/O curve (RM 2-way ANOVA: *F*_(19,513)_ = 0.871, *p* = 0.62), AMPA/NMDA ratio (CTL = 5.85 ± 0.52, LBN = 5.95 ± 0.85; *p* = 0.91; two-tailed Student’s *t*-test), or PPR (CTL = 1.37 ± 0.05, LBN = 1.33 ± 0.07; *p* = 0.64; two-tailed Student’s *t*-test; Figures 2J–N). Overall, female LBN mice only showed increased glutamate release probability in the DMS with no other synaptic impairments observed.

### 3.3. Fragmented maternal care early in life differentially affected synaptic transmission in a sex-dependent manner

In the DMS, AMPA/NMDA ratio showed a trend towards significant LBN condition × sex interaction (2-way ANOVA: *F*_(1,57)_ = 3.776, *p* = 0.057) and a significant main effect of sex (2-way ANOVA: *F*_(1,57)_ = 15.003, *p* < 0.001; Supplementary Figure 1A). Sex comparisons for PPR measures in the DMS revealed a significant LBN condition × sex interaction (2-way ANOVA: *F*_(1, 62)_ = 11.05, *p* < 0.01). Sidak’s multiple comparison test revealed that PPR for male LBN mice was significantly increased compared to female LBN mice (Male LBN vs. female LBN; *t* = 2.93 ± 0.08; *p* < 0.05; Supplementary Figure 1B).

For sex comparisons in the DLS, AMPA/NMDA ratio showed no significant LBN condition × sex interaction (2-way ANOVA: *F*_(1,57)_ = 0.205, *p* = 0.65) but a significant main effect of sex (2-way ANOVA: *F*_(1,57)_ = 41.3, *p* < 0.0001), suggesting that AMPA/NMDA ratio in the DLS differs between sexes but is not affected by ELA (Supplementary Figure 1C). Sex comparisons for PPR measures in the DLS revealed a significant LBN condition × sex interaction (2-way ANOVA: *F*_(1,64)_ = 11.31, *p* < 0.01) and a significant main effect of LBN condition (2-way ANOVA: *F*_(1,64)_ = 8.08, *p* < 0.01). Sidak’s multiple comparison test revealed that PPR for male LBN mice was significantly increased compared to all other groups (Supplementary Figure 1D) [Male CTL vs. Male LBN (*t* = 4.81 ± 0.09; *p* < 0.0001); Male LBN vs. Female CTL (*t* = 3.24 ± 0.09; *p* < 0.05); Male LBN vs. Female LBN (*t* = 3.47 ± 0.1; *p* < 0.01)].

### 3.4. Fragmented maternal care early in life impaired execution of goal-directed strategy in response to outcome devaluation in male mice

**Figure 3:**
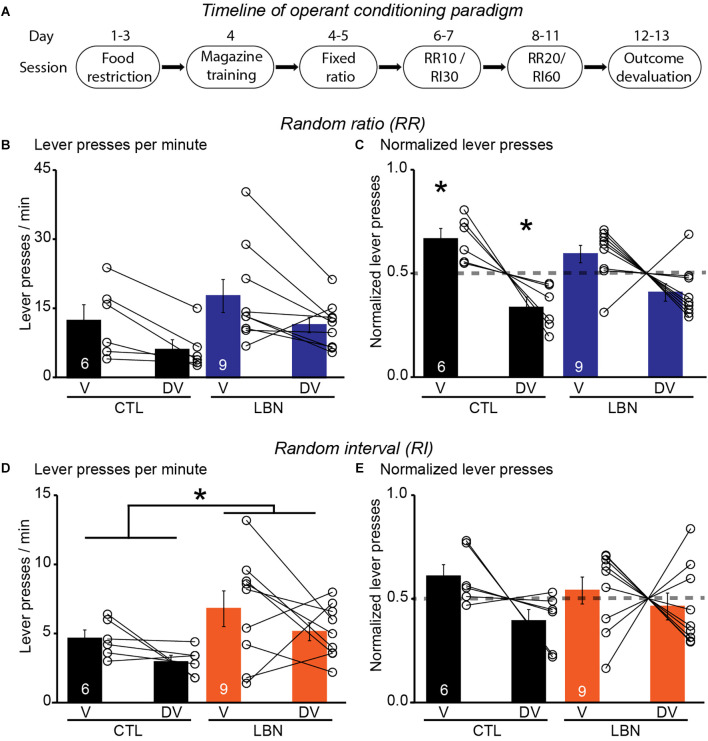
Male LBN mice failed to differentially distribute their lever presses in response to outcome devaluation in a context that promotes goal-directedness. **(A)** Experimental timeline of operant conditioning paradigm in mice. **(B,C)** Male LBN mice failed to differentially distribute their lever presses between valued (V) and devalued (DV) days in the RR context. **(B)** Summary graph of response rate (lever presses/min) on V and DV days. **(C)** Summary graph of normalized lever presses showing distribution of lever presses between V and DV days. **(D,E)** Male LBN mice exhibited normal habitual responding behavior in response to outcome devaluation in the RI context. **(D)** Summary graph of response rate (lever presses/min) on valued (V) and devalued (DV) days. **(E)** Summary graph of normalized lever presses showing distribution of lever presses between V and DV days. Data is represented as means ± SEM. Number of mice is listed inside the bar graphs. Each open circle in the summary graphs represents each mouse. Statistical assessments were performed by RM two-way ANOVA **(B,D)** by comparing male LBN to CTL mice with *p < 0.05 or one-sample t-test **(C,E)** by comparing normalized lever presses within each group of mice against a “no devaluation” point of 0.5 with *p < 0.05.

It was previously shown that LBN mice relied on habitual-action strategies in response to changes in reward value upon extensive instrumental overtraining in a context that promotes goal-directedness (Xu et al., 2022). However, previous studies did not investigate the flexibility of LBN mice to alternate between goal-directed and habitual action strategies under differing schedules of reinforcement delivery. To this end, the LBN mice underwent RR (to promote goal-directedness) and RI (to promote habitual behavior) schedules of reinforcement in two separate contexts (Figure 3A). During the conditioning phase in the RR context, the male LBN mice did not exhibit any changes in total number of lever presses [RM 2-way ANOVA: *F*_(1,13)_ = 0.7166, *p* = 0.4126] (Supplementary Figure 2A), or response rate (i.e., lever presses/min) [RM 2-way ANOVA: *F*_(1,13)_ = 0.9662, *p* = 0.3436] (Supplementary Figure 2B), or reward rate (i.e., rewards/min) [RM 2-way ANOVA: *F*_(1,13)_ = 0.2468, *p* = 0.6276] (Supplementary Figure 2C) in comparison to male CTL mice, indicating normal instrumental performance in male LBN mice. There was no significant day × LBN condition interaction for either total number of lever presses [RM 2-way ANOVA: *F*_(8,104)_ = 0.4029, *p* = 0.9167) or response rate [RM 2-way ANOVA: *F*_(8,104)_ = 0.5365, *p* = 0.8266] or reward rate [RM 2-way ANOVA: *F*_(8,104)_ = 0.2567, *p* = 0.9781]. During the outcome devaluation test, repeated measures ANOVA detected a significant effect of devaluation state [RM 2-way ANOVA: *F*_(1,13)_ = 11.05, *p* < 0.01] but not that of LBN condition [RM 2-way ANOVA: *F*_(1,13)_ = 2.195, *p* = 0.1623] on lever-pressing behavior (Figure 3B). There was also no significant ELA condition × devaluation state interaction [RM 2-way ANOVA: *F*_(1,13)_ = 0.0014472, *p* = 0.9702] in the RR context, indicating that both male CTL and LBN mice responded similarly to the effects of outcome devaluation on lever pressing behavior. However, a one sample t-test (against chance or 0.5) of normalized lever presses between valued and devalued states in the RR context showed that male CTL (valued state = 0.67, devalued state = 0.33; *p* < 0.05; one sample t-test), but not LBN mice (valued state = 0.59, devalued state = 0.41; *p* = 0.0623; one sample t-test), exhibited a higher preference for lever-pressing in valued vs. devalued state, indicating adoption of goal-directed strategy in male CTL, but not in LBN, mice (Figure 3C).

During the conditioning phase in the RI context, the male LBN mice did not exhibit any changes in total number of lever presses [RM 2-way ANOVA: *F*_(1,13)_ = 1.627, *p* = 0.2244] (Supplementary Figure 2D), response rate [RM 2-way ANOVA: *F*_(1,13)_ = 1.447, *p* = 0.2505] (Supplementary Figure 2E), or reward rate [RM 2-way ANOVA: *F*_(1,13)_ = 0.6728, *p* = 0.4269] (Supplementary Figure 2F), in comparison to CTL mice, indicating normal instrumental performance in male LBN mice. There was no significant day × LBN condition for total number of lever presses [RM 2-way ANOVA: *F*_(8,104)_ = 0.8654, *p* = 0.5482], response rate [RM 2-way ANOVA: *F*_(8,104)_ = 1.254, *p* = 0.2757], or reward rate [RM 2-way ANOVA: *F*_(8,104)_ = 0.2228, *p* = 0.9861] in the RI context. During the outcome devaluation test, repeated measures ANOVA detected no significant effect of devaluation state [RM 2-way ANOVA: *F*_(1,13)_ = 3.106, *p* = 0.1015], but a significant effect of LBN condition on lever-pressing behavior [RM 2-way ANOVA: *F*_(1,13)_ = 5.604, *p* < 0.05], indicating that male LBN mice pressed the lever significantly more than male CTL mice (Figure 3D). Despite the increase in lever pressing behavior in male LBN mice, the LBN condition × devaluation state interaction was not statistically significant in the RI context [RM 2-way ANOVA: *F*_(1,13)_ = 0.0002461, *p* = 0.9877]. A one sample t-test (against chance or 0.5) of normalized lever presses between valued and devalued states in the RI context showed that both male CTL (valued state = 0.61, devalued state = 0.39; *p* = 0.1092; one sample t-test) and LBN mice (valued state = 0.54, devalued state = 0.46; *p* = 0.5679; one sample t-test) exhibited similar distribution of lever presses between valued and devalued states (Figure 3E), indicating that both male CTL and LBN mice adopted habitual action strategy in the RI context. For the outcome devaluation procedure, male CTL and LBN mice were able to distinguish between RI and RR schedules, as depicted by altered sensitivity to differential feedback functions produced by both these schedules (Supplementary Figure 2G).

### 3.5. Fragmented maternal care early in life did not impair alternation between goal-directed and habitual action strategies in response to outcome devaluation in female mice

**Figure 4:**
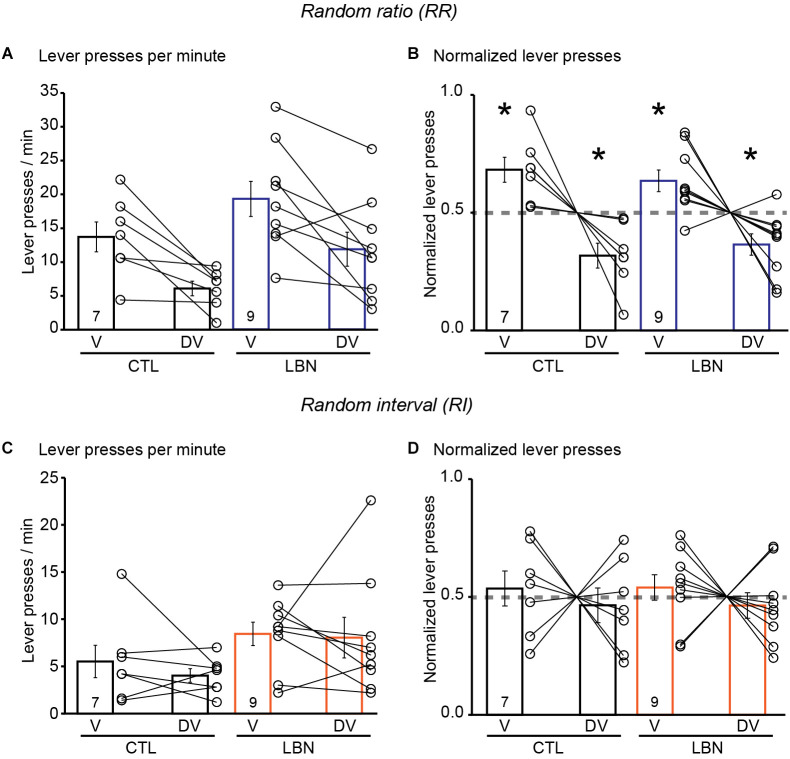
Female LBN mice did not exhibit impairments in action selection strategy in response to outcome devaluation. **(A,B)** Female LBN mice exhibited goal-directed behavior in response to outcome devaluation in the RR context. **(A)** Summary graph of response rate (lever presses/min) on V and DV days. **(B)** Summary graph of normalized lever presses showing distribution of lever presses between V and DV days. **(C,D)** Female LBN mice exhibited normal habitual responding behavior in response to outcome devaluation in the RI context. **(C)** Summary graph of response rate (lever presses/min) on valued (V) and devalued (DV) days. **(D)** Summary graph of normalized lever presses showing distribution of lever presses between V and DV days. Data is represented as means ± SEM. Number of mice is listed inside the bar graphs. Each open circle in the summary graphs represents each mouse. Statistical assessments were performed by RM two-way ANOVA **(A,C)** or one-sample t-test **(B,D)** by comparing normalized lever presses within each group of mice against a “no devaluation” point of 0.5 with *p < 0.05.

During the conditioning phase in the RR context, the female LBN mice did not exhibit any significant changes in the total number of lever presses [RM 2-way ANOVA: *F*_(1,5)_ = 0.5045, *p* = 0.4892] and there was no significant day × LBN condition interaction [RM 2-way ANOVA: *F*_(8,112)_ = 0.8015, *p* = 0.6025] (Supplementary Figure 3A). The female LBN mice exhibited an increase in response rate [RM 2-way ANOVA: *F*_(1,14)_ = 8.938, *p* < 0.01] (Supplementary Figure 3B) in comparison to female CTL mice indicating increased instrumental responding in the female LBN mice in a context that promotes goal-directed behavior. There was also a significant day × LBN condition interaction for response rate [RM 2-way ANOVA: *F*_(8, 112)_ = 2.262, *p* < 0.05] with Sidak’s *post-hoc* test confirming that female LBN mice exhibited an increase in response rate in RR10 on day 6 (*t* = 4.097, *p* < 0.05) and RR20 on day 11 (*t* = 3.877, *p* < 0.05; Supplementary Figure 3B). Female LBN mice also exhibited an increase in reward rate [RM 2-way ANOVA: *F*_(1,14)_ = 6.539, *p* < 0.05] in comparison to CTL mice without any significant day × LBN condition interaction [RM 2-way ANOVA: *F*_(8,112)_ = 1.316, *p* = 0.2428](Supplementary Figure 3C). During the outcome devaluation test, repeated measures ANOVA detected a significant effect of devaluation state [RM 2-way ANOVA: *F*_(1, 14)_ = 20.33, *p* < 0.001], and a non-significant trend towards effect of LBN condition [RM 2-way ANOVA: *F*_(1,14)_ = 4.053, *p* = 0.0637] on lever-pressing behavior. There was no significant LBN condition × devaluation state interaction [RM 2-way ANOVA: *F*_(1,14)_ = 0.002149, *p* = 0.9637], indicating a similar response in female CTL and LBN mice to the effects of outcome devaluation (Figure 4A). A one-sample t-test (against chance or 0.5) of normalized lever presses between valued and devalued states in the RR context showed that female CTL (valued state = 0.68, devalued state = 0.32; *p* < 0.05; one sample t-test) and LBN mice (valued state = 0.63, devalued state = 0.37; *p* < 0.05; one sample t-test) exhibited differential distribution of lever presses between valued and devalued states, indicating that ELA did not impair goal-directed behavior in female mice (Figure 4B).

During the conditioning phase in the RI context, the female LBN mice exhibited an increase in total number of lever presses [RM 2-way ANOVA: *F*_(1,14)_ = 8.471, *p* < 0.05] in comparison to female CTL mice (Supplementary Figure 3D). There was a significant day × LBN condition interaction for total number of lever presses [RM 2-way ANOVA: *F*_(8,112)_ = 2.979, *p* < 0.01], with Sidak’s *post-hoc* test confirming that female LBN mice exhibited a significant increase in total number of lever presses in RI30 on day 6 (*t* = 3.368, *p* < 0.05) and RI60 on day 11 (*t* = 4.477, *p* < 0.01; Supplementary Figure 3D). The female LBN mice exhibited an increase in response rate [RM 2-way ANOVA: *F*_(1,14)_ = 8.911, *p* < 0.01] in comparison to female CTL mice (Supplementary Figure 3E) indicating increased instrumental responding in female LBN mice in a context that promotes habitual behavior. There was also a significant day × LBN condition interaction for response rate [RM 2-way ANOVA: *F*_(8, 112)_ = 3.305, *p* < 0.01] with Sidak’s *post-hoc* test confirming that female LBN mice exhibited an increase in response rate in RI30 on day 6 (*t* = 3.959, *p* < 0.05) and RI60 on day 11 (*t* = 4.380, *p* < 0.01; Supplementary Figure 3E). There were no significant changes in reward rate [RM 2-way ANOVA: *F*_(1,14)_ = 0.4076, *p* = 0.5335] between the female LBN and CTL mice (Supplementary Figure 3F). Repeated measures ANOVA detected a non-significant trend towards effect of LBN condition [RM 2-way ANOVA: *F*_(1,14)_ = 3.399, *p* = 0.0865] and no effect of devaluation state [RM 2-way ANOVA: *F*_(1,14)_ = 0.5006, *p* = 0.4909] on lever-pressing behavior (Figure 4C) in RI context. There was also no significant LBN condition × devaluation state interaction [RM 2-way ANOVA: *F*_(1,14)_ = 0.1659, *p* = 0.6899]. A one-sample t-test (against chance or 0.5) of normalized lever presses between valued and devalued states in the RI context showed that both the female CTL (valued state = 0.54, devalued state = 0.46; *p* = 0.6349; one sample t-test) and LBN mice (valued state = 0.54, devalued state = 0.46; *p* = 0.4793; one sample t-test) were insensitive to outcome devaluation as evidenced by their equal distribution of lever presses between valued and devalued states (Figure 4D). For the outcome devaluation procedure, female CTL and LBN mice were able to distinguish between RI and RR schedules, as depicted by altered sensitivity to differential feedback functions produced by both these schedules (Supplementary Figure 3G).

### 3.6. Fragmented maternal care early in life induced behavioral inflexibility in a reversal learning paradigm

**Figure 5:**
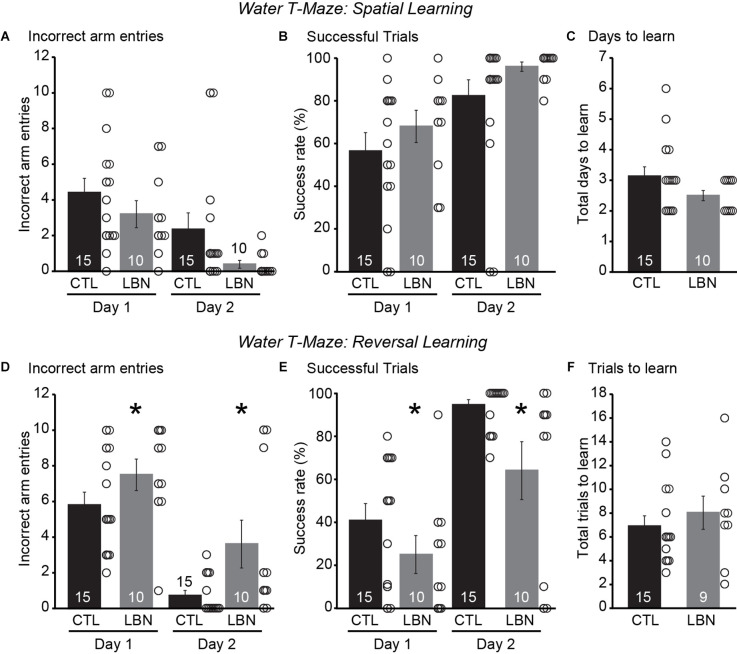
LBN mice exhibited impaired behavioral flexibility in a water T-maze reversal learning paradigm. **(A–C)** LBN mice exhibited normal spatial navigation. **(A)** Summary graph of incorrect arm entries during the spatial task on days 1 and 2. **(B)** Summary graph of percentage of successful trials during the spatial task on days 1 and 2. **(C)** Summary graph of total number of days taken to learn the spatial task. **(D–F)** LBN mice exhibited reversal learning deficits. **(D)** Summary graph of incorrect arm entries during reversal learning task on days 1 and 2. **(E)** Summary graph of percentage of successful trials during the reversal learning task on days 1 and 2. **(F)** Summary graph of total number of trials taken to learn the reversal task. Data is represented as means ± SEM. Number of mice is listed inside the bar graphs. Each open circle in the summary graphs represents each mouse. Statistical assessments were performed by RM two-way ANOVA **(A,B,D,E)** or two-tailed Student’s *t*-test **(C,F)** by comparing CTL to LBN mice with *p < 0.05.

During the spatial navigation phase, repeated measures ANOVA detected a significant effect of day [RM 2-way ANOVA: *F*_(1,23)_ = 23.31, *p* < 0.0001] but not that of LBN condition [RM 2-way ANOVA: *F*_(1,23)_ = 2.234, *p* = 0.15] on the number of incorrect arm entries as well as on the % of successful trials [RM 2-way ANOVA: Day: *F*_(1,23)_ = 23.31, *p* < 0.0001; LBN condition: *F*_(1,23)_ = 2.234, *p* = 0.15], indicating normal spatial navigation in LBN mice (Figures 5A,B). There was also no significant day × LBN condition interaction for number of incorrect arm entries [RM 2-way ANOVA: *F*_(1,23)_ = 0.3357, *p* = 0.57] or % of successful trials [RM 2-way ANOVA: *F*_(1,23)_ = 0.3357, *p* = 0.57]. The LBN mice tended to take fewer number of days to learn the task of finding the platform in comparison to CTL mice (CTL = 3.133 ± 0.31 days, LBN = 2.5 ± 0.17 days; *p* = 0.08; two-tailed Student’s *t*-test; Figure 5C).

During the reversal learning phase, there was a significant effect of LBN condition [RM 2-way ANOVA: *F*_(1,23)_ = 5.284, *p* < 0.05] and day [RM 2-way ANOVA: *F*_(1,23)_ = 71.89, *p* < 0.0001] on number of incorrect arm entries and on % of successful trials [RM 2-way ANOVA: LBN condition: *F*_(1,23)_ = 5.102, *p* < 0.05; Day: *F*_(1,23)_ = 71.00, *p* < 0.0001] indicating reversal learning deficits in LBN mice across both the days of the test (Figures 5D,E). There was also no significant day × LBN condition interaction for number of incorrect arm entries [RM 2-way ANOVA: *F*_(1,23)_ = 1.340, *p* = 0.26] and % of successful trials [RM 2-way ANOVA: *F*_(1,23)_ = 1.436, *p* = 0.24], indicating that ELA impaired reversal learning throughout the entirety of the testing period. Finally, there were no significant differences in total trials to learn reversal of contingencies upon intradimensional shifts in behavior between the LBN and CTL mice (CTL = 6.8 ± 0.88 trials, LBN = 8 ± 1.39 trials; *p* = 0.48; two-tailed Student’s *t*-test; Figure 5F). Segregation of mice for water T-maze experiments by their sexes did not show significant differences in number of incorrect arm entries or success rate (%) between CTL and LBN mice within each sex for spatial navigation and reversal learning phases (Supplementary Data). ANOVA analysis also did not detect any significant effects of sex on number of incorrect arm entries or success rate (%) within the CTL and LBN group for spatial navigation and reversal learning phases (Supplementary Data). Thus, sexes were combined for water T-maze experiments.

## 4. Discussion

Our investigation provides new insights into the functional impact of ELA on excitatory synaptic transmission in the dorsal striatum and behavioral adaptability in a dynamic environment. We find that ELA can impair the ability to strategize action selection or disengage from preferred behavioral patterns to learn new ones upon changes in circumstances, in order to achieve a specific goal.

Previous examinations into the impact of ELA in adult male mice showed an overall increase in dendritic spine density and immunolabeling of PSD-95 in MSNs of the DLS (Xu et al., 2022). Here, we addressed the functional impact of ELA on corticostriatal synaptic transmission. Despite morphological studies showing an increase in excitatory synapses, our data suggest that overall excitatory transmission from cortical inputs to MSNs is decreased in male mice following LBN. An important distinction between the two studies is that the current study focused on cortical inputs specifically as they are one of the major sources of glutamate to the dorsal striatum. Two possibilities arise from our current data: (i) LBN caused a decrease in glutamate release in DLS of male mice as an adaptation response to the increased excitatory synaptic connections; or (ii) LBN has opposing effects on different sources of glutamate input to the dorsal striatum (e.g., thalamus, hippocampus). If the latter is the case, then we would expect other excitatory inputs to the dorsal striatum to show increased function. A recent study found that LBN disrupts synaptic pruning mechanisms in the hippocampus, resulting in increased dendritic arborization and c-Fos activation of CA1 pyramidal cells (Dayananda et al., 2023). Future investigations are warranted to determine how LBN impacts thalamic and hippocampal inputs to the dorsal striatum.

For all electrophysiological measures, we started by measuring the relationship between EPSC amplitude and stimulation intensity of cortical inputs (I/O curve). Measures of I/O curve in voltage clamp configuration are good indicators of overall neurotransmission from a specific input. However, due to large variability in amplitude between cells, mild impairments to synaptic transmission are unlikely to be detected in I/O measures. Thus, the current study used I/O curve as a measure of overall glutamate transmission. Impairments in the DLS of male LBN mice were interpreted as a severe impact of LBN on corticostriatal transmission. I/O curve measures, however, do not indicate if synaptic impairments are pre- or post-synaptic. Thus, we measured PPR and AMPA/NMDA ratio to directly address pre- and post-synaptic alterations, respectively. The probability of glutamate release was decreased in both DLS and DMS of male LBN mice, as indicated by the increase in PPR in both regions (the interpretation of change in PPR is inverse to direction of the change in PPR, e.g., increase in PPR = decrease in release probability). Interestingly, male LBN mice showed a significant decrease of AMPA/NMDA ratio in the DMS, but not in DLS indicating a change in the relative contribution of AMPA and NMDA receptors to overall EPSC. Closer examination of these EPSCs revealed that the difference in the AMPA/NMDA ratio for the LBN mice was due to a combination of smaller AMPA currents and larger NMDA currents. It still remains uncertain whether this shift is due to changes in NMDA receptor kinetics or if density distribution of AMPA and NMDA receptors has changed. Additionally, it is well-established that long-term plasticity in MSNs of the dorsal striatum requires NMDA receptor activation (Calabresi et al., 1992; Dang et al., 2006). Thus, changes in NMDA receptor function or density could have profound consequences for striatal plasticity. Alternatively, changes in NMDA receptor subunit composition could also result in changes in AMPA/NMDA ratio. NMDA receptor subunits 2A and 2B have been shown to play critical roles in the dorsal striatum by regulating complex motor skills and reward-seeking behaviors, respectively (Lemay-Clermont et al., 2011; Gass et al., 2018).

Excitatory and inhibitory neurotransmission operate in concert within an intact circuit to determine neuronal, and subsequently, circuit function. Our finding of impaired glutamate transmission in male LBN mice opens the possibility of an imbalance between excitatory and inhibitory transmission in the dorsal striatum. MSNs in the dorsal striatum receive GABAergic inputs from local GABAergic interneurons and adjacent MSNs (Taverna et al., 2004; Kocaturk et al., 2022). Additionally, recent work has shown that glycine receptors containing the α2 subunit are critical for regulating excitability of striatal MSNs by controlling tonic inhibition (Molchanova et al., 2018). Future investigations into ELA-induced changes in inhibition would be necessary for a more complete understanding of the ELA-induced effects in dorsal striatum. Furthermore, it is known that expression of GABA_A_ receptor subunits differs between D1- and D2-MSNs (Boccalaro et al., 2019). Therefore, it is possible that LBN effects to striatal inhibition are cell-type specific, which could have profound consequences to downstream processes when excitatory and inhibitory transmission are working together.

In contrast to the male data, female LBN mice had no impairments in glutamate transmission in the DLS. PPR measurements in the DMS revealed a significant decrease in ratio for female LBN mice, indicating that female LBN mice had a higher probability of glutamate release in the DMS. At present, the underlying reason for opposing results for PPR in the DMS between sexes is not clear. However, it is clear that sex is an important factor in the role of ELA on glutamate release in the DMS. This also appears to be the case for the nucleus accumbens (NAc) where sex-dependent effects of ELA on glutamate neurotransmission were also observed. ELA reduced the frequency of spontaneous excitatory postsynaptic currents (sEPSCs) in the NAc in male, but not in female rats (Ordoñes Sanchez et al., 2021). The sex-differences in glutamate neurotransmission were attributed to unique transcriptional signature in the NAc between male and female LBN rats (Ordoñes Sanchez et al., 2021). Similar studies evaluating the effects of ELA on gene expression in the DMS would be warranted in elucidating differences in PPR between both sexes.

ELA did not affect habitual responding as evidenced by the lack of sensitivity to outcome devaluation in both sexes in the RI context. On the other hand, male (but not female) LBN mice adopted a similar action strategy (i.e., habitual action) in response to outcome devaluation in the RR context, indicating impaired goal-directed behavior. The adoption of the same action strategy under differing contingencies of reward delivery suggests the male LBN mice are unable to utilize prior knowledge of the consequences of their actions and the value of those consequences to guide action control. The generalization of habitual behavior in male LBN mice can be linked to the previously observed increase in synaptic contacts in the DLS, but not in the DMS, in male LBN mice (Xu et al., 2022). The DMS receives projections from associative cortices including the anterior cingulate cortex and the premotor cortex (Hadjas et al., 2020), and has been implicated in regulation of goal-directed behavior (Yin et al., 2005a,b; Hart et al., 2018; Terra et al., 2020). The DLS receives projections from the sensorimotor cortex (Hadjas et al., 2020) and has been linked to habitual behaviors (Yin et al., 2004, 2006; Eskenazi and Neumaier, 2011; Jenrette et al., 2019). The increase in synaptic connections in the DLS suggests a preponderance of DLS circuitry over that of DMS in the balance between goal-directed and habitual actions. This can potentially explain the over-reliance of male LBN mice on habitual actions in circumstances that warrant goal-directedness.

The ability of female LBN mice to differentiate between goal-directed and habitual action strategies under differing contingencies of reward delivery suggests normal neural circuit development and maturation. However, the effects of LBN on dendritic differentiation and spinogenesis in the dorsal striatum of female mice have not been investigated and future investigations would be warranted to confirm this hypothesis. Interestingly, the instrumental response in the RI and RR context was significantly higher in female (but not in male) LBN mice, indicating greater proficiency in encoding the incentive value of an instrumental task and faster formation of action-outcome (A-O) associations in the female LBN mice. The faster rate of habit formation in female LBN mice could potentially underlie the induction of compulsive drug-seeking habit by ELA (Levis et al., 2021). Interestingly, in the same study, the compulsive behavior induced by ELA was specific to drugs of reward (heroin) or highly palatable food (sweet banana pellets or chocolate) but not to less salient food rewards (chow pellets; Levis et al., 2021). This findings is in line with our current study in which female LBN mice did not develop habitual behavior upon devaluation of less salient food rewards. Surprisingly, the over-reliance of male LBN mice on habitual behaviors does not seem to directly apply to the effects of ELA on predisposition to drug addiction. In studies assessing the effects of ELA on rewarding properties of drug abuse, male LBN rats exhibited behavioral deficits consistent with anhedonia (Bolton et al., 2018a,b; Levis et al., 2022). In this context, we must consider animal species differences when correlating our findings with those that assess the effects of ELA on reward-seeking behavior since majority of those studies were conducted in rats and not in mice (Bolton et al., 2018a,b; Levis et al., 2021, 2022). Furthermore, habitual and drug-seeking behaviors are controlled by two different neural circuits. Habitual behavior is governed by DLS (Yin et al., 2004, 2006) while drug reward behavior is governed by NAc, amygdala, and paraventricular nucleus (Chaudhri et al., 2010; Koob and Volkow, 2010; Millan et al., 2017). The question of how the dorsal and ventral striatum regulate such behaviors in a parallel or subsequent manner remains to be explored.

The water T-maze experiment produced a different outcome, where reversal learning deficits were observed in LBN mice without any impairments in spatial memory. The normal spatial navigation in LBN mice from our study contradicts previous studies that have reported spatial memory deficits due to alterations in hippocampal neurogenesis (Naninck et al., 2015; Bath et al., 2017), synaptic plasticity (Ivy et al., 2010), and loss of dendritic spines (Xu et al., 2022). Nevertheless, previous findings on spatial memory deficits were obtained from Morris water maze tests, which require a more complex strategy for spatial navigation than the water T-maze, which requires only a simple body movement (i.e., turning right or left into the correct arm; Bardgett et al., 2003; Guariglia et al., 2011). The opposing results from these two behavioral tasks are not surprising considering that these tests measure different aspects of behavioral flexibility. Water T-maze measures intradimensional shifts in behavior that utilize a non-probabilistic reinforcement schedule, where the correct choice is reinforced 100% of the time. In contrast, the lever pressing action in the instrumental learning task delivers the reinforcer 15% of the time. Thus, it appears that LBN induced a generalized effect in flexibility tests that requires disengagement from previously learned behavioral responses and the adoption of new responses upon intradimensional shifts in A-O contingency; and a sex-dependent effect in paradigms that requires updating behavioral responses upon devaluation of the A-O contingency.

Mechanisms of synaptic signal facilitation and depression are thought to underlie synaptic temporal dynamics that are necessary for learning and cognitive function (Fortune and Rose, 2001; Zucker and Regehr, 2002). A previous study in rodents using *in vivo* electrophysiology recordings showed that action potential frequency increases significantly in the DLS and DMS during habitual and goal-directed behaviors, respectively (Gremel and Costa, 2013). In this study, a higher firing rate of DMS neurons and a lower firing rate of DLS neurons was associated with sensitivity to changes in value of outcome in the RR context vs. the RI contexts. The decrease in glutamate release in DMS and DLS in male LBN mice could potentially mean that the MSNs would be less likely to be depolarized and generate an action potential. Given the difference in magnitude of the glutamate release impairment between DLS and DMS, it is likely that cortical inputs to one or both of these regions are not able to sustain elevated levels of glutamate release that would increase the likelihood of action potentials in MSNs. This could ultimately affect the firing rate of MSNs in the DMS and DLS, thus impairing the sensitivity of mice to outcome devaluation in a context that promotes goal-directedness. A separate study using *in vivo* recordings showed that the frequency of action potentials increased in the DLS during correct runs in a T-maze task (Smith and Graybiel, 2016). Our findings on cortical glutamate release suggest that ELA disrupts synaptic mechanisms associated with short-term plasticity. In the current study, all control mice showed paired-pulse facilitation to two electrical pulses delivered at 40 Hz. This suggests that, under normal circumstances, cortical terminals increase their glutamate release in response to elevated activation, such as what we would see during a cognitive task or learning. Additionally, all control mice showed the same magnitude of facilitation (PPR 1.2). It is likely that signal facilitation in corticostriatal synapses requires precise levels of neurotransmitter facilitation, and that impairments to short-term plasticity in either direction (increased or decreased) could result in the same cognitive impairment. Our electrophysiological results, combined with our T-maze results, agree with this observation. Future *in vivo* electrophysiological recordings of DMS and DLS in LBN mice during the instrumental learning test or T-maze would be necessary to assess a direct role for short term plasticity deficits in failure to strategize action selection. Furthermore, previous work has linked endocannabinoid signaling to the gating of strategy selection (Gremel et al., 2016), which could potentially be a candidate for a molecular mechanism of impairment in ELA. Investigations into the role of endocannabinoids in corticostriatal synaptic transmission would be warranted for future studies.

In summary, our study begins to shed light on the corticostriatal synaptic mechanisms, which are impacted by ELA, underlying the shift between goal-directed and habitual action strategies. Behavioral inflexibility in the form of impaired habitual control or deficits in goal-directedness is often associated with psychiatric disorders that are linked to ELA including schizophrenia (Morris et al., 2015), obsessive compulsive disorder (OCD; Gillan et al., 2011; Simmler and Ozawa, 2019; Dong et al., 2020), and drug addiction (Goldstein and Volkow, 2011; Belin et al., 2013). These findings will lay the foundation for the development of novel therapeutic strategies for the treatment of behavioral inflexibility in individuals who are diagnosed with ELA-associated psychiatric disorders.

## Data availability statement

The original contributions presented in the study are included in the article/Supplementary material, further inquiries can be directed to the corresponding author.

## Ethics statement

The animal study was reviewed and approved by IACUC committee, University of California, Irvine.

## Author contributions

Conceptualization and design, writing and editing: SK, GC, and LYC. Methodology: SK and GC. Data analysis: SK, MH, and GC. All authors contributed to the article and approved the submitted version.
