# Supplementary material for: Early life adversity impaired dorsal striatal synaptic transmission and behavioral adaptability to appropriate action selection in a sex-dependent manner
Source: Front Synaptic Neurosci. 2023 Apr 5;15:1128640. doi: 10.3389/fnsyn.2023.1128640 (PMC10116150; doi:10.3389/fnsyn.2023.1128640)
Supplement: Supplementary file 1 [file Presentation_1.pdf]

Supplementary Material

Early life adversity impaired dorsal striatal synaptic transmission and behavioral adaptability to appropriate action selection in a sex-dependent manner

Gregory de Carvalho†, Sheraz Khoja†, Mulatwa T. Haile, Lulu Y. Chen*

*Correspondence: Dr. Lulu Y. Chen: chenly@uci.edu

† These authors contributed equally and share the first authorship.

1. Supplementary Data

This document comprises of seven spreadsheets. The first five spreadsheets contain raw values and statistical tests for Fig. 1-5 respectively. The sixth and seventh spreadsheets contain raw values and statistical tests for supplementary figures S1 & S2.

2. Supplementary figures

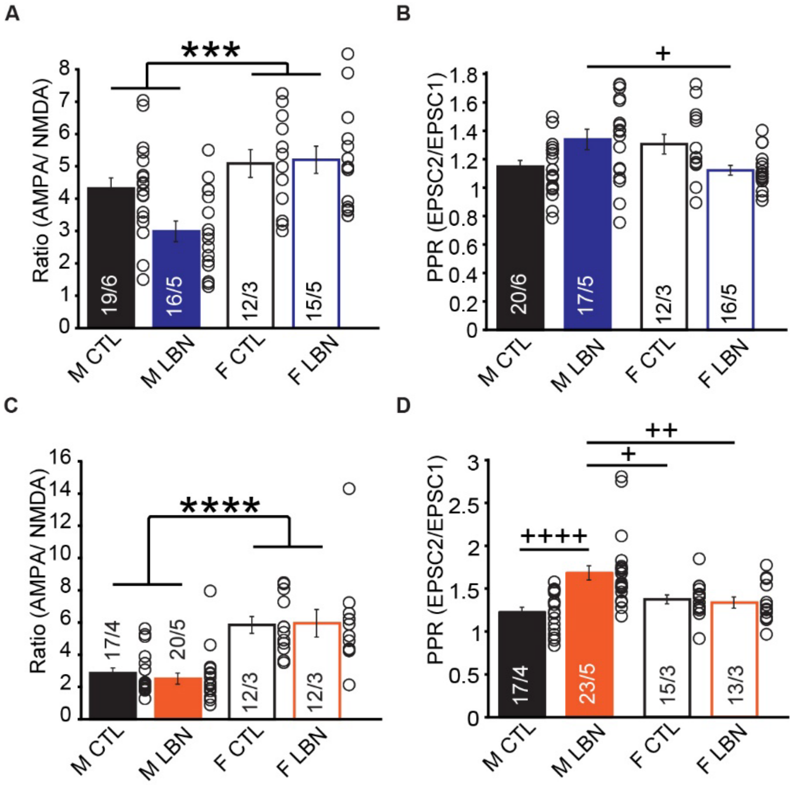

Supplementary Figure 1. LBN affects synaptic transmission in a sex-dependent manner.

(A) Summary graph of two-way ANOVA test of AMPA/NMDA ratio in the DMS for male and female mice.

(B) Summary graph of two-way ANOVA test of PPR in the DMS for male and female mice.

(C) Summary graph of two-way ANOVA test of AMPA/NMDA ratio in the DLS for male and female mice.

(D) Summary graph of two-way ANOVA test of PPR in the DLS for male and female mice.

Data is represented as means ± SEM. Number of neurons/mice are listed inside (or on top) of the bar graphs. Each open circle in the summary graphs represents the average of each recorded cell. Statistical assessments were performed by two-way ANOVA by comparing male and female (LBN and CTL) mice with ***$p < 0.001$, ****$p < 0.0001$. Significance of Sidak's multiple comparison test (for tests where LBN x Sex interaction was significant) is shown as +$p < 0.05$, ++$p < 0.01$, and ++++$p < 0.0001$

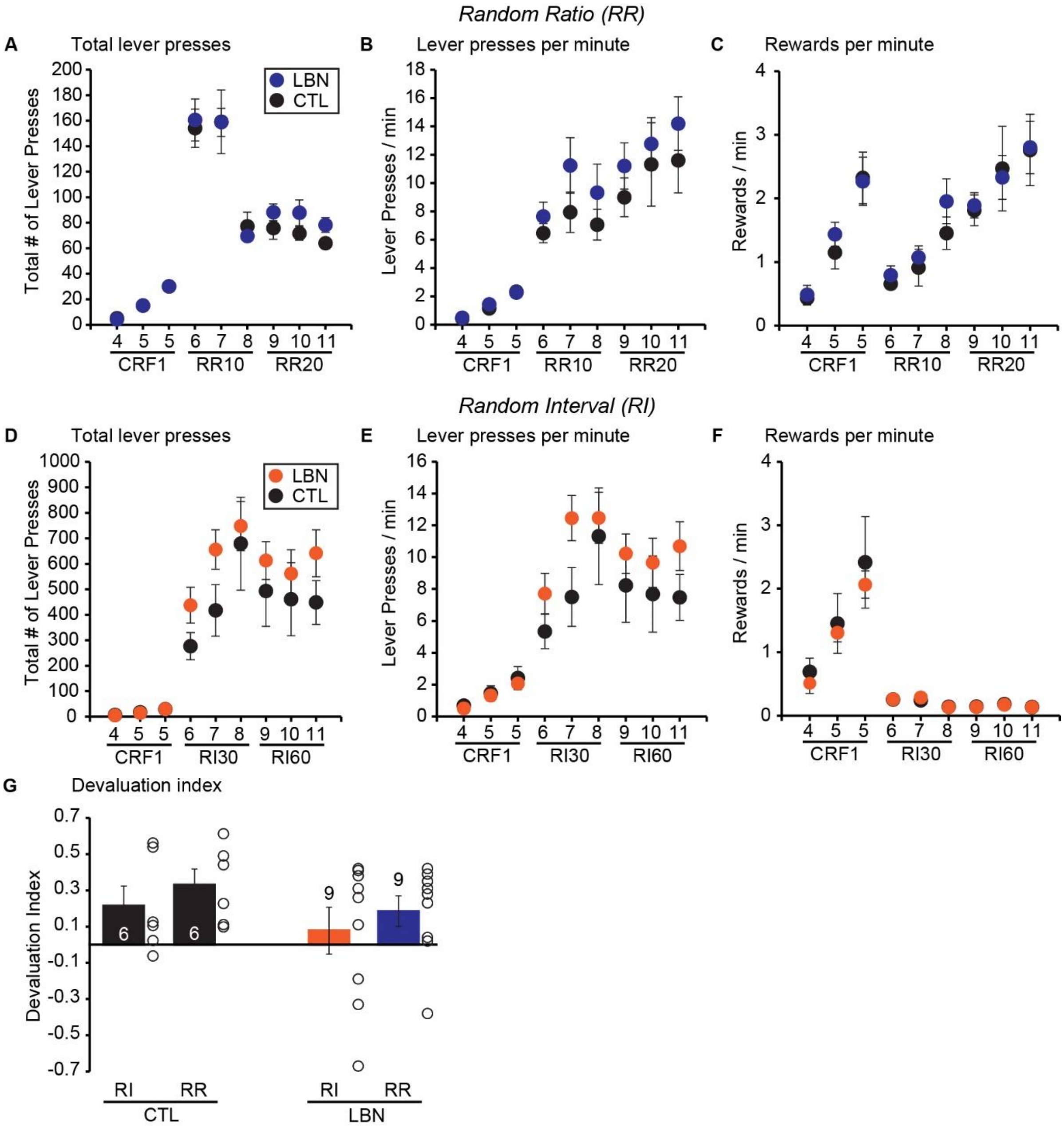

Supplementary Figure 2. Male LBN mice demonstrated normal instrumental performance in the RI and RR contexts.

(A-C) Male LBN mice exhibited normal instrumental responding in the RR context. **(A)** time-course plot showing total number of lever presses. **(B)** time-course plot showing response rate (lever presses/min). **(C)** time-course plot showing reward rate (rewards/min).

(D-F) Male LBN mice exhibited normal instrumental responding in the RI context. **(D)** time-course plot showing total number of lever presses. **(E)** time-course plot showing response rate (lever presses/min). **(F)** time-course plot showing reward rate (rewards/min).

(G) Summary graph of devaluation index between male CTL and LBN mice in the RI and RR contexts.

Each circle in the time-course plots **(A-F)** represents means ± SEM for 6 male CTL and 9 male LBN mice. The data is represented as means ± SEM and number of mice is listed inside the bar graphs and each open circle in the summary graph represents each mouse **(G).** Statistical assessments were performed by RM two-way ANOVA **(A-F)**.

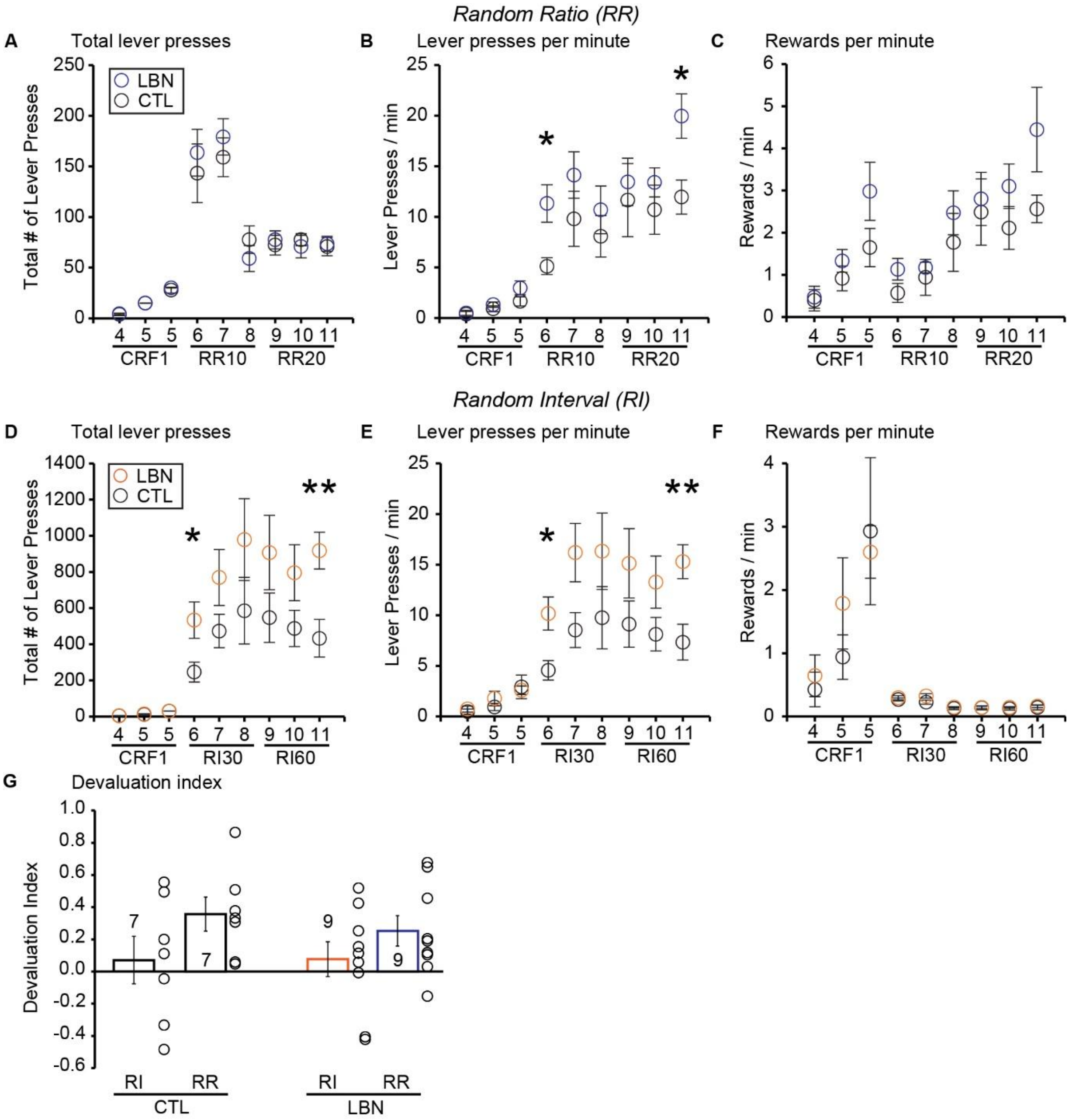

Supplementary Figure 3. Female LBN mice demonstrated increased instrumental responding in the RI and RR contexts.

(A-C) Female LBN mice exhibited increased response rate and reward rate in the RR context. **(A)** time-course plot showing total number of lever presses. **(B)** time-course plot showing response rate (lever presses/min). **(C)** time-course plot showing reward rate (rewards/min).

(D-F) Female LBN mice exhibited increased response rate that was accompanied by a greater number of lever presses in the RI context. **(D)** time-course plot showing total number of lever presses. **(E)** time-course plot showing response rate (lever presses/min). **(F)** time-course plot reward rate (rewards/min).

(G) Summary graph of devaluation index between female CTL and LBN mice in the RI and RR contexts.

Each circle in the time-course plot represents means ± SEM for 7 female CTL and 9 LBN mice. The data is represented as means ± SEM and number of mice is listed inside the bar graphs and each open circle in the summary graph represents each mouse **(G).** Statistical assessments were performed by RM two-way ANOVA followed by Sidak's *post hoc* test **(A-F)** by comparing female LBN to CTL mice with *$p < 0.05$, ** $p < 0.01$.
